# Integrated Transcriptomic Profiling Reveals Distinct and Overlapping Transcriptional Responses and Regulatory Pathways Mediated by Salicylic Acid, Jasmonic Acid, and Abscisic Acid in Lotus (*Nelumbo nucifera*)

**DOI:** 10.3390/ijms27177957

**Published:** 2026-09-07

**Authors:** Junyang Xu, Ziyan Yang, Ji Yang, Yanyan Meng, Xinqiong Liu

**Affiliations:** School of Life Sciences, South-Central Minzu University, Wuhan 430074, China; 202421141135@mail.scuec.edu.cn (J.X.); 202421141064@mail.scuec.edu.cn (Z.Y.); w992995886@gmail.com (J.Y.)

**Keywords:** *Nelumbo nucifera*, transcriptome sequencing, stress response, shared transcriptional responses, hub genes

## Abstract

Sacred lotus (*Nelumbo nucifera*) is an important aquatic crop whose growth and productivity are severely constrained by environmental stresses. Salicylic Acid (SA), Jasmonic Acid (JA), and Abscisic Acid (ABA) are key plant growth regulators (PGRs) involved in stress responses, but their regulatory mechanisms in lotus remain unclear. In this study, transcriptome sequencing was performed in lotus seedlings treated with exogenous SA, JA, and ABA to characterize hormone-responsive regulatory networks. SA predominantly resulted in transcriptional repression, with responsive genes mainly associated with photosynthesis and ribosome-related pathways, whereas JA and ABA showed similar regulatory patterns with enrichment of hormone signaling and Mitogen-activated protein kinase (MAPK) pathways, but distinct roles in defense regulation and stress adaptation. A total of 607 genes were identified as commonly responsive to the three PGRs and were significantly enriched in cold response, defense response, secondary metabolism, and photosynthesis-related pathways. Protein–protein interaction analysis identified two hub genes encoding light-harvesting chlorophyll a/b-binding proteins, suggesting that the photosynthesis–antenna proteins pathway may represent a convergent regulatory node in hormone-mediated stress responses. This study provides new insights into SA-, JA-, and ABA-mediated stress responses and identifies potential candidate genes for improving stress tolerance in lotus.

## 1. Introduction

*Nelumbo nucifera*, a perennial aquatic herb of the family Nelumbonaceae, is not only a traditional ornamental flower in China but also possesses exceptional ornamental, economic, cultural, and ecological value [1,2]. Currently, *Nelumbo nucifera* is extensively cultivated and utilized worldwide. However, under both natural and cultivated conditions, *Nelumbo nucifera* is frequently subjected to adverse environmental factors, including extreme temperatures (heat and cold), salinity stress, heavy metal contamination, and drought stress. These abiotic stresses significantly inhibit the growth and development of *Nelumbo nucifera*, manifesting as leaf chlorosis and wilting, as well as petal senescence and abscission. Moreover, such stresses disrupt the endogenous redox homeostasis, impair the photosynthetic apparatus, and compromise cellular structural integrity. These detrimental effects severely constrain the overall yield, quality, and economic efficiency of *Nelumbo nucifera* production [1,3,4]. Therefore, systematically elucidating the molecular mechanisms underlying stress tolerance regulation in *Nelumbo nucifera* holds substantial theoretical and practical significance for the innovation of stress-resilient germplasm and the optimization of cultivated traits in this species.

Over the course of long-term evolution, plants have developed sophisticated regulatory networks for stress response. Among the core components of these networks, PGRs serve as essential signaling molecules that play indispensable roles in mediating plant responses to both biotic and abiotic stresses [5,6,7]. Extensive studies have demonstrated that ABA, SA, and JA, along with their signaling interactions, constitute a crucial molecular basis for plant defense against both biotic stresses (such as pathogen infection) and abiotic stresses [5,7]. Specifically, the JA signaling pathway primarily mediates plant immune defense response against necrotrophic fungi and other pathogens, enhancing plant resistance through the activation of downstream defense-related gene expression [8]; in contrast, SA mainly orchestrates the defense response against biotrophic pathogens and is capable of inducing the establishment of systemic acquired resistance (SAR) [9]. Beyond its classical function as a quintessential “stress hormone”—whereby it induces stomatal closure to reduce transpirational water loss under drought conditions—ABA also indirectly participates in plant defense against biotic stress by restricting microbial entry through stomata [10]. Collectively, these three PGRs perform distinct yet interconnected roles within the plant stress regulatory network. Through distinct yet partially overlapping signaling responses, these hormones contribute to the coordinated regulation of plant growth and development with stress defense. This finely tuned regulatory interplay constitutes a core mechanism underlying plant adaptation to complex and fluctuating environments [7,11].

For the optimal growth and long-term survival of plants, the precise regulation of the defense response is of paramount importance. This requires not only the efficient activation of specific defense pathways tailored to particular stressors but also the avoidance of excessive energy expenditure on ineffective defense measures. Furthermore, it necessitates the delicate balancing of resource allocation between defense response, basal growth metabolism, and concurrent responses to other environmental challenges [11,12]. This regulatory process involves distinct and partially overlapping hormonal signaling responses, which may contribute to the regulation of plant growth, development, and stress defense. This phenomenon is collectively referred to as hormone crosstalk [11,13]. Hormone crosstalk not only constitutes a core component of the plant immune signaling network but also, beyond enabling the fine-tuning and balancing of the defense response, enhances the specificity of induced defense reactions toward distinct biotic interactors and reinforces the robustness of stress responses. As such, it represents a key mechanism underlying plant adaptation to complex environmental conditions [7,14].

Existing studies have provided preliminary evidence that ABA, SA, and JA may participate in the stress response processes of *Nelumbo nucifera*. For instance, exogenous SA participates in the defense response to soft rot disease caused by *Pectobacterium carotovorum* (syn. *Erwinia carotovora*) in multiple plant species via the TGA2–LNC88940–miR528–SOD regulatory module [15]. JA has been demonstrated to modulate the response of *Nelumbo nucifera* to complete submergence through the regulation of ethylene response factors (ERFs) [16]; furthermore, ABA can improve the adaptability of *Nelumbo nucifera* rhizomes to salinity stress by inducing the expression of salt tolerance-associated genes, such as those encoding bZIP transcription factors [17]. However, current research on hormonal regulation in *Nelumbo nucifera* has predominantly focused on the response of a single hormone to a single stress condition. Consequently, several core scientific questions remain largely unexplored and lack systematic investigation and in-depth elucidation. These questions include: how SA, JA, and ABA—three key defense PGRs—orchestrate global transcriptional reprogramming in *Nelumbo nucifera*; what similarities and differences exist among the signaling pathways activated by each hormone; and whether functional crosstalk occurs among these hormonal pathways. This knowledge gap significantly constrains a comprehensive understanding of the stress-resilience signaling network in *Nelumbo nucifera*.

Based on this, the present study utilized seedlings of *Nelumbo nucifera* as experimental material and subjected them to exogenous treatments with SA, ABA, and JA, respectively. RNA-sequencing (RNA-seq) profiling was employed to comprehensively characterize the global gene expression patterns of seedlings of *Nelumbo nucifera* under SA, JA, and ABA treatments. Through bioinformatics analysis, we systematically compared the differentially expressed genes (DEGs), enriched functional pathways, and regulatory modes among the three treatments. Furthermore, hormone-specific responsive genes and core genes co-regulated by multiple PGRs were identified. This study aims to address the following core scientific questions: (1) How do SA, ABA, and JA specifically regulate the transcriptome expression profiles of seedlings of *Nelumbo nucifera*? (2) What are the similarities and specificities among the signaling pathways activated by the three PGRs? (3) Are there common key genes or hormone-specific responsive genes involved in the responses to the three PGRs, and do potential nodes of hormonal crosstalk exist among them? The findings of this study will provide systematic insights into the molecular mechanisms by which SA, ABA, and JA orchestrate stress responses in *Nelumbo nucifera*. Furthermore, this work will establish a crucial molecular foundation for the in-depth elucidation of the stress-resilience signaling network in *Nelumbo nucifera*. Concomitantly, the results will offer both a theoretical framework and vital genetic resources for the future enhancement of stress tolerance and yield in *Nelumbo nucifera*, whether achieved through genetic improvement strategies or chemical regulatory interventions.

## 2. Results

### 2.1. Quality Control and Genome Alignment Analysis of Transcriptome Sequencing Data

**Figure 1:**
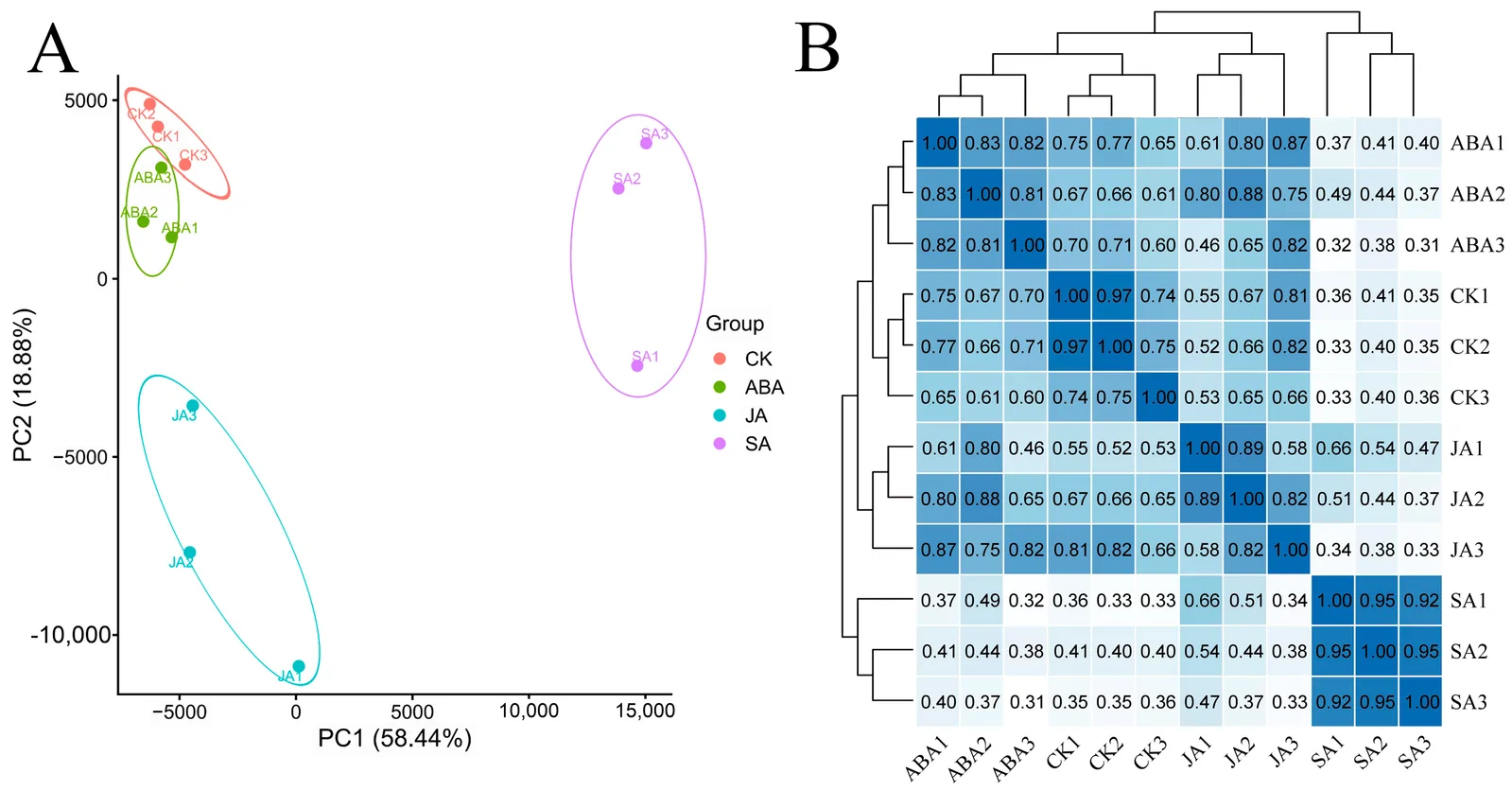
Principal component analysis (PCA) and sample correlation analysis of RNA-seq samples. (**A**) Principal component analysis (PCA) of all RNA-seq samples. Samples from the same treatment group are indicated by the same color. (**B**) Pairwise sample correlation heatmap showing the correlation among RNA-seq samples.

Principal component analysis (PCA) and sample correlation analysis showed that biological replicates within each treatment exhibited high consistency and clustered closely. The treatment groups displayed distinct overall transcriptional patterns, supporting the reliability and reproducibility of the RNA-seq data (Figure 1). The overall quality of the sequencing data was excellent (Supplementary Table S1), yielding a total of 69.24 Gb of clean data. The data efficiency rate for all samples exceeded 94.11%, with Q20 base percentages of no less than 99.29% and Q30 base percentages of no less than 96.11%. The GC content remained stable, ranging from 44.78% to 45.79%, exhibiting no obvious base bias. These results met the requirements for subsequent bioinformatics analysis.

The clean reads from each sample were aligned to the reference genome of *Nelumbo nucifera*. The results showed (Supplementary Table S2) that the genome mapping reads ranged from 94.37% to 96.98%, with sample J3 exhibiting the highest mapping reads and sample S3 showing the lowest but still exceeding 94%. The total reads per sample ranged from 37.16 million to 47.71 million, with an average of 42.13 million mapped reads. The multiple mapped reads ranged from 1.6% to 2.58%, and the unique mapped reads ranged from 91.8% to 95.33%. These results indicated a high degree of concordance between the sequencing reads and the reference genome, with no obvious contamination, thereby demonstrating strong data reliability. Following transcript reconstruction based on the obtained data, a total of 1433 novel genes of *Nelumbo nucifera* were identified, among which 1298 genes received valid functional annotations, yielding an annotation rate of 90.58%. This provides new gene resources for subsequent functional studies of genes in *Nelumbo nucifera*.

### 2.2. Screening and Pathway Analysis of Differentially Expressed Genes in Nelumbo nucifera Seedlings Under Single-Hormone Treatments

Using the control check (CK) group as the control, significant DEGs were screened for the SA, JA, and ABA treatment groups, respectively. Combined with Gene Ontology (GO) functional annotation and Kyoto Encyclopedia of Genes and Genomes (KEGG) pathway enrichment analysis, the specific regulatory patterns of different PGRs on gene expression in *Nelumbo nucifera* seedlings were elucidated.

#### 2.2.1. Comparison Between ABA Group and Mock Group

**Figure 2:**
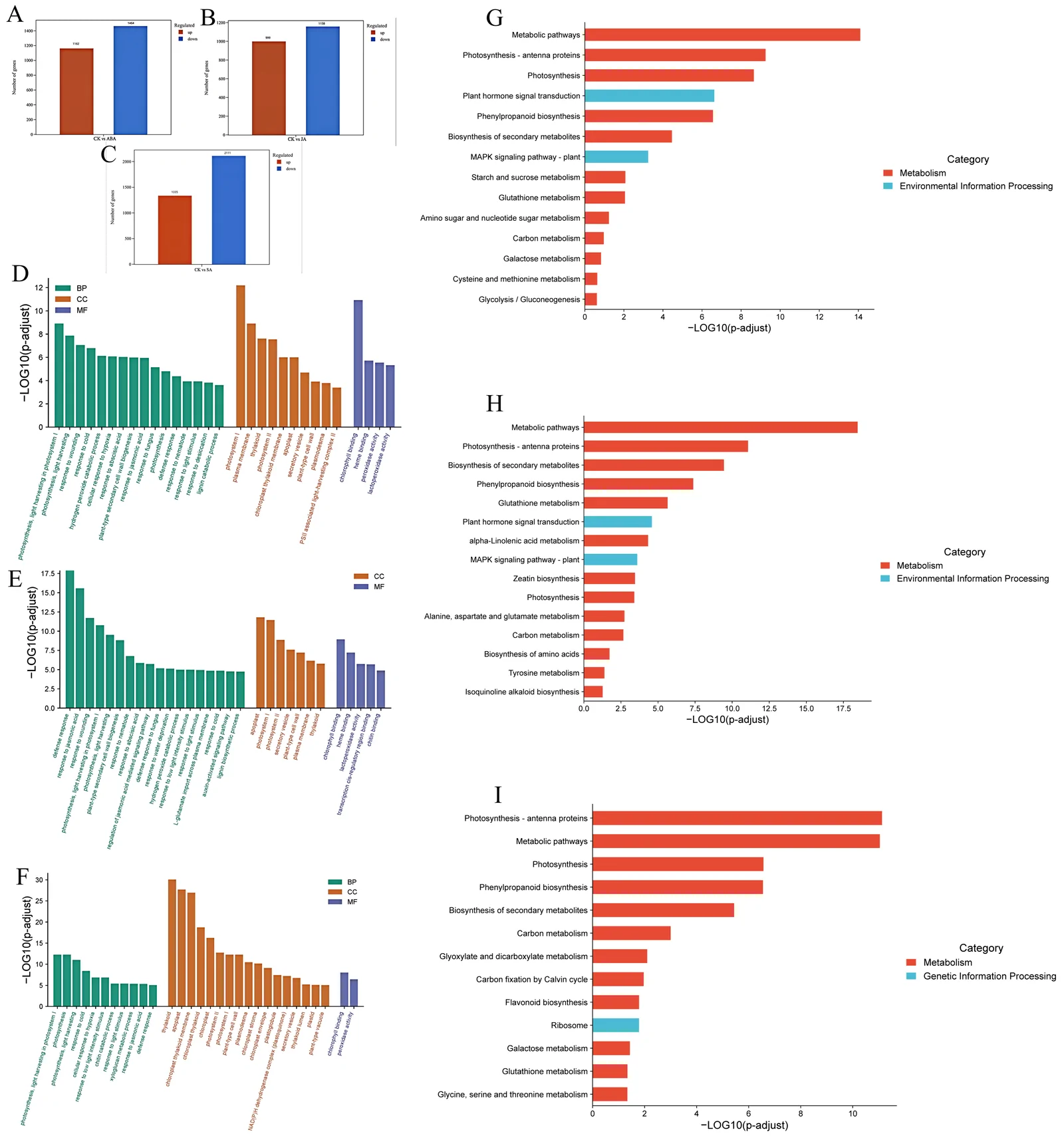
Analysis of differentially expressed genes (DEGs) under single-hormone treatments of Salicylic Acid (SA), Jasmonic Acid (JA), and Abscisic Acid (ABA). (**A**) DEGs upon ABA treatment; (**B**) DEGs upon JA treatment; (**C**) DEGs upon SA treatment; (**D**) three primary Gene Ontology (GO) category enrichment of DEGs under ABA treatment; (**E**) three primary GO category enrichment of DEGs under JA treatment; and (**F**) three primary GO category enrichment of DEGs under SA treatment. (**G**) Kyoto Encyclopedia of Genes and Genomes (KEGG) pathway enrichment of DEGs under ABA treatment; (**H**) KEGG pathway enrichment of DEGs under JA treatment; and (**I**) KEGG pathway enrichment of DEGs under SA treatment.

Compared with the control group, a total of 2626 DEGs were detected in *Nelumbo nucifera* seedlings under ABA treatment, including 1162 upregulated genes and 1464 downregulated genes (Figure 2A). GO enrichment analysis (Figure 2D; Supplementary Table S6) revealed that ABA-responsive DEGs were predominantly enriched in photosynthesis-related biological-process terms. Photosystem-I-associated terms dominated cellular-component enrichment, and chlorophyll binding represented the top enriched molecular-function term. These observations suggest that ABA primarily modulates photosystem-I-associated photosynthetic metabolism in lotus seedlings, without broadly affecting other photosynthetic structures, which distinguishes ABA from SA-mediated photosynthetic reprogramming. KEGG enrichment further showed that metabolic pathways constituted the major proportion of enriched terms under ABA treatment. Similar to JA, DEGs were enriched in photosynthesis–antenna proteins, photosynthesis, plant hormone signal transduction, and the plant MAPK signaling pathway (Figure 2G; Supplementary Table S7). These shared enriched pathways imply potential common stress-signal-response modules between ABA and JA.

#### 2.2.2. Comparison Between JA Group and Mock Group

Compared with the control group, a total of 2157 DEGs were detected in *Nelumbo nucifera* seedlings under JA treatment, including 999 upregulated genes and 1158 downregulated genes (Figure 2B). GO enrichment analysis (Figure 2E; Supplementary Table S6) revealed that JA-triggered DEGs showed prominent enrichment for defense-response terms in the biological-process category, supporting the central role of JA in driving defense-associated transcriptional reprogramming [18,19,20]. Enrichment was also observed for apoplast and photosystem-I-related terms in the cellular-component category, as well as chlorophyll-binding terms in the molecular-function category, pointing to concurrent modulation of photosynthetic-metabolism-related gene expression by JA. KEGG analysis further identified enrichment of metabolic pathways, plant hormone signal transduction, and plant MAPK signaling among the JA-responsive genes (Figure 2H; Supplementary Table S7). Together, these results indicate that JA treatment is associated with transcriptional changes involving defense responses, photosynthesis-related functions, metabolism, and stress-related signaling in Nelumbo nucifera seedlings.

#### 2.2.3. Comparison Between SA Group and Mock Group

Compared with the control group, a total of 3446 DEGs were detected in *Nelumbo nucifera* seedlings under SA treatment, including 1135 upregulated genes and 2111 downregulated genes (Figure 2C). GO enrichment analysis (Figure 2F; Supplementary Table S6) showed prominent enrichment for photosynthetic-structure-associated cellular-component terms, including thylakoid, apoplast and chloroplast thylakoid membrane. Biological-process terms related to photosynthesis and molecular-function term chlorophyll binding were also enriched. KEGG pathway analysis (Figure 2I; Supplementary Table S7) indicated that SA-responsive DEGs were mainly assigned to metabolic pathways and photosynthesis–antenna proteins. In addition, the ribosome pathway was enriched among SA-responsive genes. These results indicate that SA treatment is associated with transcriptional changes involving photosynthetic functions, metabolism, and translation-related processes in Nelumbo nucifera seedlings.

### 2.3. Analysis of DEGs Shared Between Individual PGR Treatments in Pairwise Comparisons

To characterize transcriptional responses shared among hormone treatments, DEGs identified in the pairwise comparisons were examined for overlap. Functional enrichment analyses of shared genes were further performed separately for co-upregulated and co-downregulated genes to distinguish shared transcriptional induction from repression (Supplementary Tables S8 and S9).

**Figure 3:**
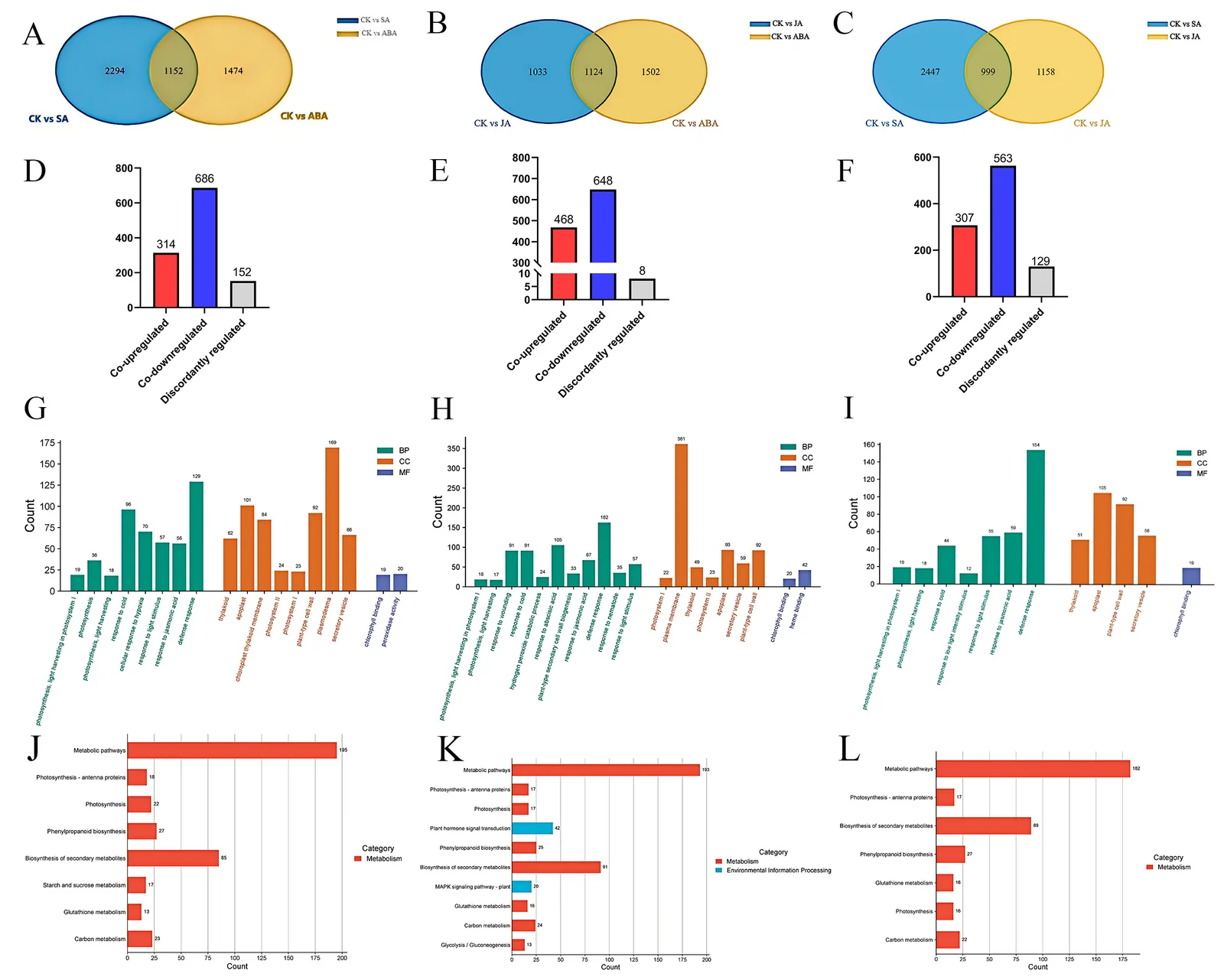
Analysis of co-responsive differentially expressed genes (DEGs) to SA, JA and ABA. (**A**) Venn diagram of co-responsive DEGs between SA and ABA treatments; (**B**) Venn diagram of co-responsive DEGs between JA and ABA treatments; (**C**) Venn diagram of co-responsive DEGs between SA and JA treatments; and (**D**–**F**) classification of the shared DEGs into co-upregulated, co-downregulated, and discordantly regulated genes for SA–ABA, JA–ABA, and SA–JA treatment pairs, respectively. (**G**–**I**) GO enrichment analysis of the shared DEGs for SA–ABA, JA–ABA, and SA–JA treatment pairs, respectively. (**J**–**L**) KEGG pathway enrichment analysis of the shared DEGs for SA–ABA, JA–ABA, and SA–JA treatment pairs, respectively.

Compared with the control group, a total of 999 DEGs were co-responsive to SA and JA (Figure 3C). These DEGs were mainly enriched in defense-response (biological process), apoplast (cellular component), and chlorophyll-binding (molecular function) terms (Figure 3I; Supplementary Table S8), pointing to overlapping transcriptional responses related to defense and photosynthetic metabolism under SA and JA treatments. The apoplast may serve as a key cellular compartment for these shared transcriptional outputs. KEGG enrichment showed that metabolic pathways remained dominant among SA–JA co-responsive DEGs (Figure 3L; Supplementary Table S9), consistent with observations from individual-hormone treatments. A total of 1152 DEGs were co-responsive to SA and ABA (Figure 3A), with top enriched terms including defense response, plasmodesma and peroxidase activity (Figure 3G; Supplementary Table S8). Enrichment of peroxidase-activity-related genes implies that SA–ABA shared transcriptional programs are largely oriented toward oxidative-stress defense. A total of 1124 DEGs were co-responsive to JA and ABA (Figure 3B). These genes were enriched in defense-response, plasma-membrane and heme-binding terms (Figure 3H; Supplementary Table S8). Enrichment of plasma-membrane-related annotations suggests that shared transcriptional responses of JA and ABA are associated with transmembrane perception and transduction of stress signals. Co-responsive DEGs of JA and ABA were also enriched in plant hormone signal transduction and plant MAPK signaling pathway (Figure 3K; Supplementary Table S9), which implies potential cross-regulatory relationships at the transcriptional level between JA- and ABA-dependent gene programs. Across the pairwise comparisons of hormone-responsive genes, defense response was a major enriched biological process, suggesting that SA, JA, and ABA treatments share transcriptional responses associated with stress defense. SA–JA focused on defense response and photosynthetic metabolism, SA–ABA focused on oxidative stress defense, and JA–ABA focused on transmembrane signal perception and transmission. Furthermore, among the pairwise comparisons, JA- and ABA-responsive genes were the only group enriched in hormone signal transduction pathways, highlighting a prominent shared transcriptional response associated with hormone signaling.

### 2.4. Analysis of Differentially Expressed Genes and Pathways Co-Responsive to the Three PGRs

**Figure 4:**
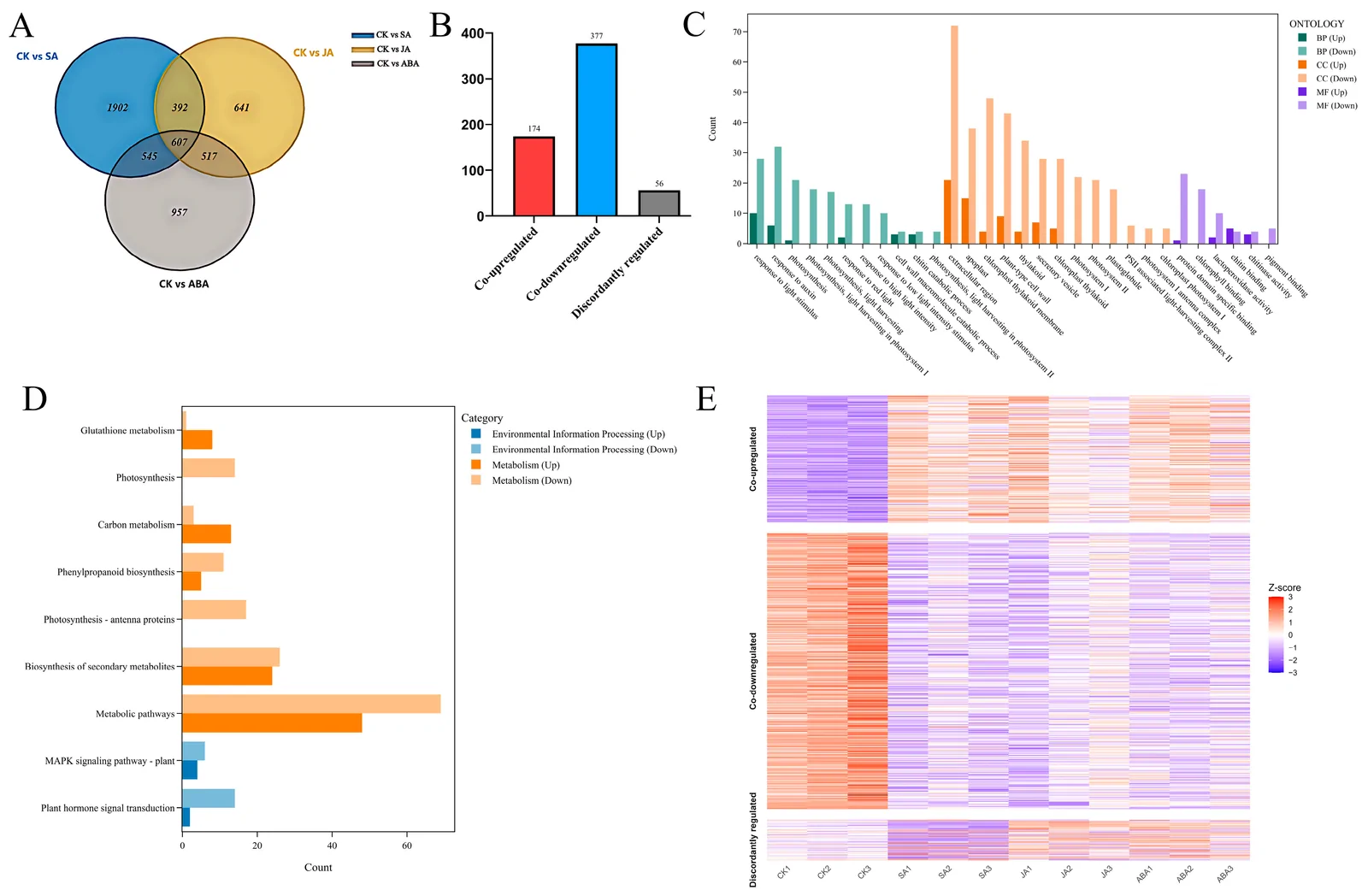
Analysis of DEGs co-responsive to SA, JA and ABA. (**A**) Venn diagram showing the shared DEGs among SA, JA, and ABA treatments; (**B**) classification of shared DEGs according to their expression patterns; (**C**) GO enrichment analysis of shared DEGs; (**D**) KEGG enrichment analysis of shared DEGs; and (**E**) expression-pattern heatmap of shared DEGs.

Compared with the control group, a total of 607 DEGs were identified as commonly responsive to salicylic acid (SA), jasmonic acid (JA), and abscisic acid (ABA) treatments (Figure 4A). The number of co-responsive DEGs was substantially lower than that identified under each individual hormone treatment, suggesting that the three PGRs induced both shared and treatment-specific transcriptional responses in Nelumbo nucifera seedlings. Based on their expression patterns across the three hormone treatments, the 607 co-responsive genes were further classified into three groups: co-upregulated, co-downregulated, and discordantly regulated genes, comprising 174, 377, and 56 genes, respectively (Figure 4B). The heatmap further illustrated the shared and distinct expression patterns of these three groups across the SA, JA, and ABA treatments (Figure 4E). GO enrichment analysis showed that these co-responsive genes were mainly associated with biological processes related to cold response and defense response, as well as cellular components including the apoplast and plant-type cell wall (Figure 4C; Supplementary Table S10). These enriched terms indicate that the shared transcriptional responses induced by the three PGR treatments were primarily associated with stress-responsive processes, particularly cold stress and defense-related functions.

KEGG enrichment demonstrated that co-responsive genes concentrated in metabolic and stress-related pathways (Figure 4D; Supplementary Table S11). Besides general metabolic pathways and biosynthesis of secondary metabolites, prominent enriched pathways included photosynthesis–antenna proteins, phenylpropanoid biosynthesis, carbon metabolism, photosynthesis, and glutathione metabolism. Plant hormone signal transduction and plant MAPK signaling pathway were also enriched. It should be noted that pathway enrichment only reflects over-representation of DEGs within given functional categories and cannot serve as direct evidence for actual pathway activation or magnitude of regulatory effects. These collective transcriptional signatures point to coordinated changes in metabolism, photosynthesis, secondary metabolism and stress-response programs upon SA, JA and ABA exposure in *N. nucifera* seedlings.

In addition, we systematically identified transcription factors from the transcriptome of *Nelumbo nucifera* seedlings. A total of 960 transcription factors belonging to 85 transcription factors families were identified in each of the SA, JA and ABA treatment groups, and the transcription factors’ family compositions as well as the gene number within each family remained consistent across the three hormone treatments. The most abundant transcription factors families included Homeobox (102), Zf (95), AP2/EREBP (71), bHLH (70), bZIP (65), MYB (51), NAC (47) and WRKY (32), all of which have been widely reported as key transcription factor families involved in plant hormone signal transduction and abiotic/biotic stress responses. These results indicate that SA, JA and ABA treatments can all activate a broad transcription factor regulatory network in lotus seedlings, and that the three hormones share a highly similar response profile at the transcriptional factor level.

### 2.5. Construction of Protein–Protein Interaction (PPI) Network and Identification of Hub Genes for Co-Responsive Genes

**Figure 5:**
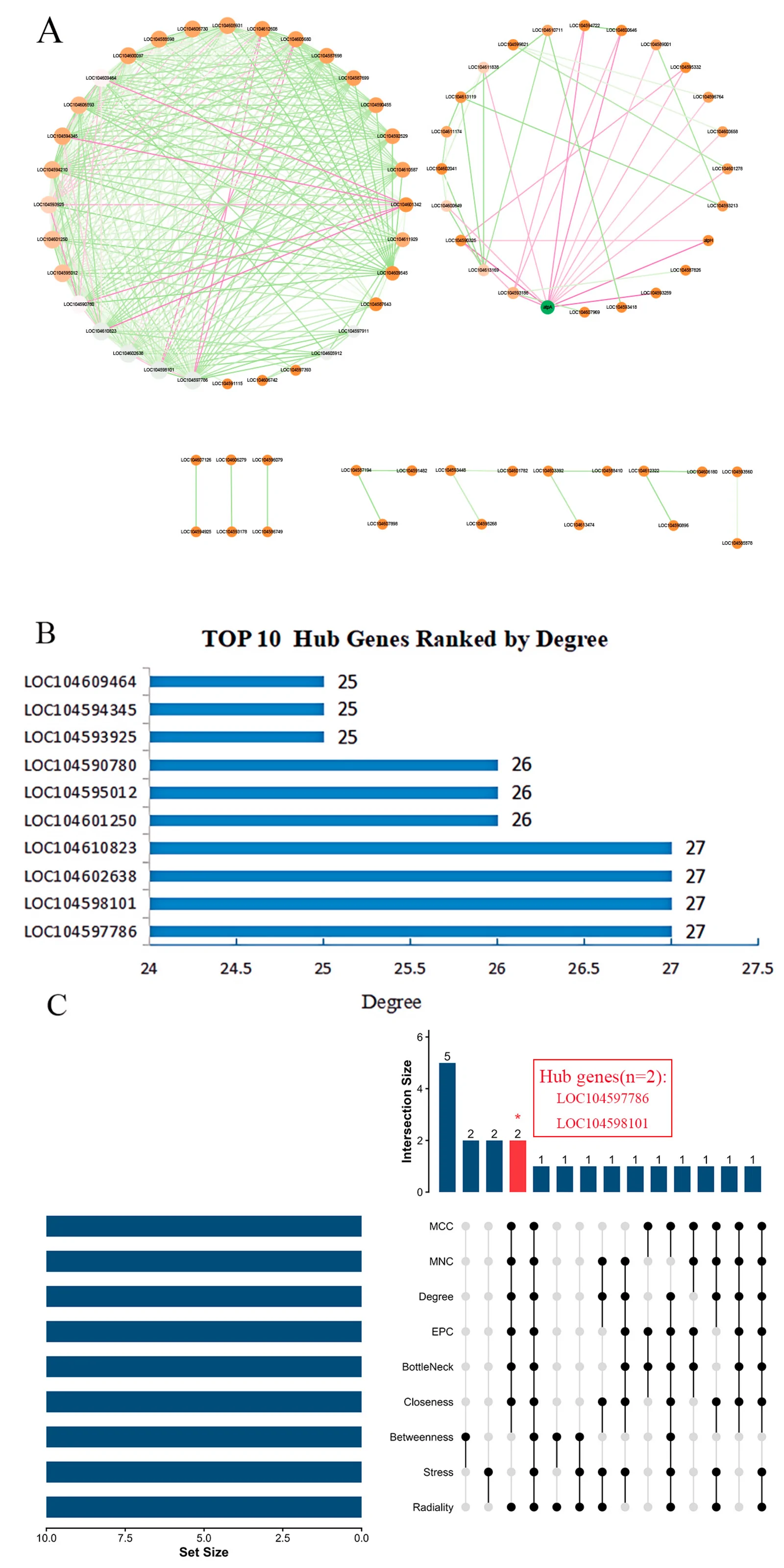
Construction of protein–protein interaction (PPI) network and identification of hub genes for co-responsive genes. (**A**) PPI network of co-responsive genes; (**B**) top 10 hub genes ranked by degree centrality in the PPI network. The degree represents the number of interactions for each gene; (**C**) UpSet plot illustrating the intersections of hub genes predicted by nine topological analysis algorithms of the cytoHubba plugin in Cytoscape, including MCC, MNC, Degree, EPC, Closeness, Betweenness, BottleNeck, Stress and Radiality. The top 10 genes were screened from each algorithm, and two overlapping genes (*LOC104597786*, *LOC104598101*) were identified as the final hub genes. Red asterisks indicate the two identified hub genes.

To further explore the key regulators co-responsive to the three PGRs, protein–protein interaction (PPI) network analysis was performed on the 607 co-DEGs. The minimum interaction confidence score was set to 0.7. Isolated nodes were removed prior to network export and visualization (Figure 5A). The resulting network displayed dense predicted inter-gene connections derived from STRING database output. Multiple members within the same gene family exhibited predicted association; these in silico associations do not constitute experimental evidence for physical protein-complex formation. Several subsets of genes formed linear predicted connection patterns within the network. Such topological arrangements are purely computational predictions and cannot be interpreted as proof of real-world sequential signal-transmission cascades in lotus seedlings.

For hub-gene detection, topological analyses were performed via the cytoHubba plugin. The top 10 ranked genes from nine independent algorithms were retrieved (Supplementary Table S5), and overlapping candidates were displayed in an UpSet plot (Figure 5C). Two genes (*LOC104597786* and *LOC104598101*) were repeatedly ranked highly by multiple topological algorithms and designated as computationally predicted hub candidates. Among them, *LOC104597786*, *LOC104598101*, *LOC104602638* and *LOC104610823* showed prominent values in degree centrality analysis (Figure 5B), indicating their core roles within the network.

### 2.6. Validation of Transcriptome Sequencing Results by Quantitative Real-Time PCR(qRT-PCR)

**Figure 6:**
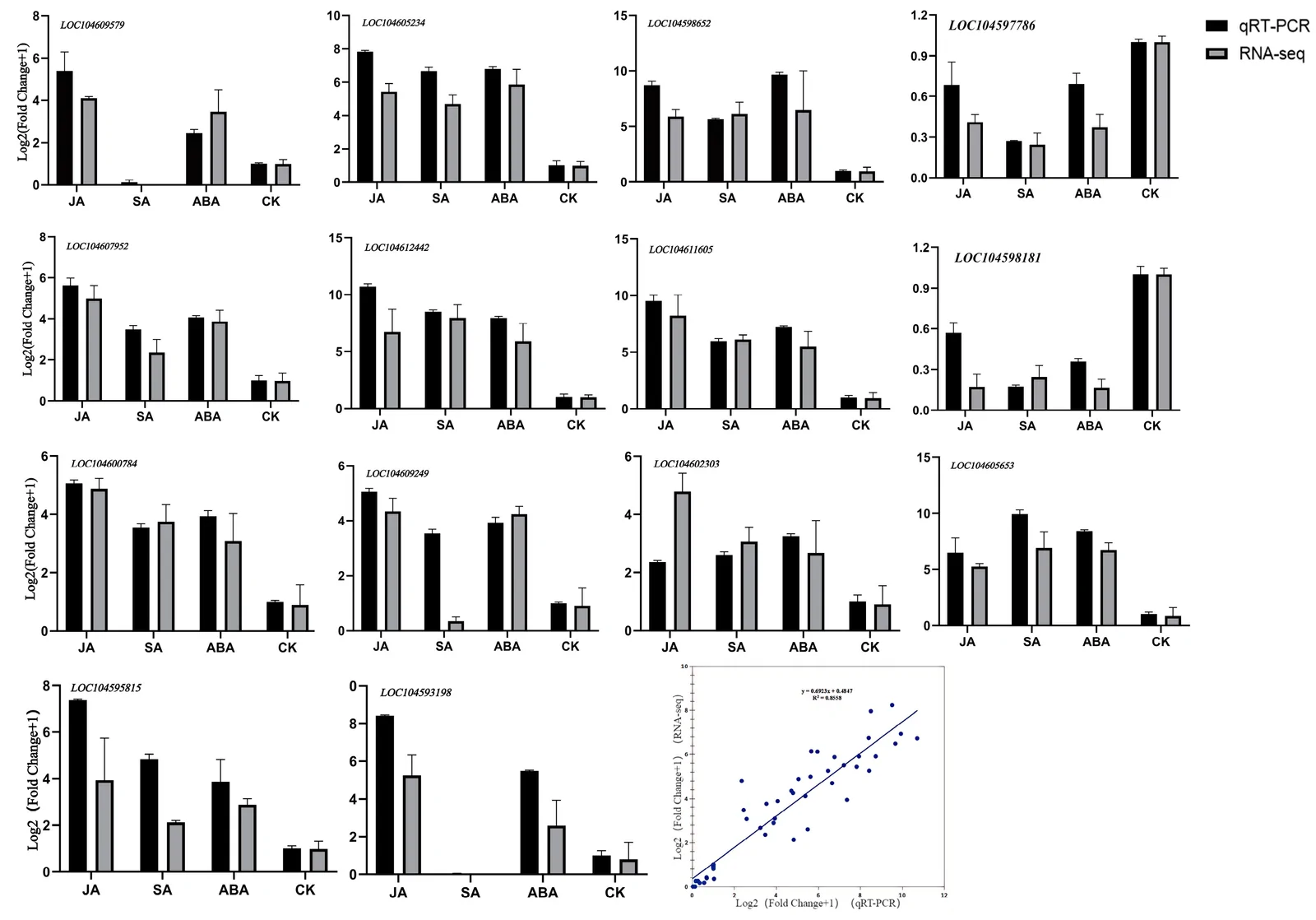
Validation of RNA-seq results via quantitative real-time PCR (qRT-PCR). Fourteen representative DEGs were selected for expression verification in *Nelumbo nucifera* seedlings under CK, SA, JA and ABA treatments. Bar graphs show relative expression levels detected by qRT-PCR, and line graphs represent gene expression levels obtained from RNA-seq. The consistent expression trends between the two methods confirm the reliability of the transcriptome data.

To validate the reliability of the RNA-seq data, 14 genes were randomly selected from the DEGs for qRT-PCR verification. The results showed (Figure 6) that the expression trends of all candidate genes in each hormone treatment group were largely consistent with the RNA-seq results. Correlation analysis yielded a strong positive correlation (R^2^ = 0.8558), confirming the high reliability of our transcriptome data for subsequent molecular-mechanism investigation.

It is worth noting that three of the twelve validated genes (*LOC104609579*, *LOC104609249*, and *LOC104593198*) showed lower expression levels under SA treatment compared with the CK control group. As a multifunctional plant growth regulator, SA not only induces numerous defense-related genes but also represses the transcription of a subset of genes, which represents an important feature of SA-mediated transcriptional reprogramming. The SA-triggered down-regulation of these genes may reflect a growth-defense trade-off strategy in lotus: plants repress certain transcripts to reallocate cellular substances and energy toward stress defense. In addition, the expression trends of these three genes measured by qRT-PCR are consistent with those obtained from RNA-seq, further supporting the reliability of our transcriptomic data.

## 3. Discussion

Currently, research on the molecular mechanisms by which plant PGRs regulate growth and development has predominantly focused on model plants such as *Arabidopsis thaliana* and rice [21,22,23,24], whereas transcriptomic studies investigating the shared transcriptional responses to multiple hormones in *Nelumbo nucifera*, a distinctive aquatic economic crop, remain limited. This study systematically elucidated the differentially expressed gene profiles and transcriptional regulatory characteristics of *Nelumbo nucifera* seedlings under exogenous SA, JA, and ABA treatments, providing a theoretical foundation for revealing the molecular mechanisms underlying the responses of *Nelumbo nucifera* to exogenous PGRs.

In Arabidopsis, SA-mediated stress responses also redistribute resources between growth and defense by repressing the expression of photosynthesis-related genes [14,25,26]. SA’s core strategy in mediating stress responses in lotus was mainly associated with transcriptional changes in photosynthesis-, metabolism-, and translation-related processes [15]. Although previous studies have shown that exogenous SA treatment can enhance resistance to soft rot disease in lotus, the present transcriptomic data do not directly establish the molecular mechanism underlying this phenotype. The transcriptional regulatory patterns of JA and ABA exhibited partially overlapping patterns. DEGs responsive to both JA and ABA were enriched in gene sets related to stress-signal transduction and amplification, pointing to the potential involvement of plant hormone signal transduction and MAPK signaling modules [27,28]. This is consistent with existing research showing that JA mediates submergence stress responses and pathogen defense in *Nelumbo nucifera* [16]. Judging from DEG profiles, ABA modulates a narrower scope of photosynthetic pathways relative to SA, targeting only the photosystem I core complex without involving processes such as ribosomal translation. Compared with SA-responsive genes, ABA-responsive genes showed a more limited representation of photosynthesis- and translation-related functions in the present dataset, consistent with the established role of ABA in plant stress responses [10]. Because the treatments were performed independently, these observations should therefore be interpreted as hormone-associated transcriptional signatures rather than direct evidence of pathway activation or hormonal crosstalk. Combined-hormone treatments and functional experiments will be required to test these potential interactions in *Nelumbo nucifera*.

Plant hormones can interact to coordinate plant responses to complex environmental conditions [29,30,31]. JA and ABA shared DEGs were enriched in hormone signal transduction-related pathways. This observation is consistent with previous studies reporting interactions between JA and ABA in abiotic stress responses in model plants [32]. Notably, the apoplast was the most significantly enriched cellular component in both SA–JA co-responsive genes and the three-hormone common responsive genes. Previous studies have confirmed that the apoplast serves as a central compartment for intercellular signaling, ROS homeostasis regulation, and defense responses in plants [33,34]. Plant PGRs achieve specific crosstalk among signaling pathways and form complex and sophisticated transcriptional regulatory networks by participating in different functional regulatory modules and mediating multidimensional transcriptional regulation and molecular interactions [10,32,35]. Collectively, shared transcriptional programs across SA, JA and ABA converge upon photosynthesis-, metabolism- and defense-related physiological processes, which may support lotus adaptation to both biotic and abiotic stresses. Secondary-metabolite-related pathways are among these shared responses, consistent with the well-documented roles of phenolic and terpenoid metabolites in plant stress resilience [36,37]. Highly similar transcription-factor profiles across hormone treatments imply that *Nelumbo nucifera* employs a conserved transcriptional toolkit when perceiving distinct hormone signals, with the bHLH family standing out as a promising candidate for future stress-resistance research. Collectively, these transcriptomic signatures suggest SA, JA and ABA do not operate in complete isolation but may form interconnected regulatory networks to facilitate stress adaptation and offer candidate resources for stress-resistance breeding in *Nelumbo nucifera*.

**Figure 7:**
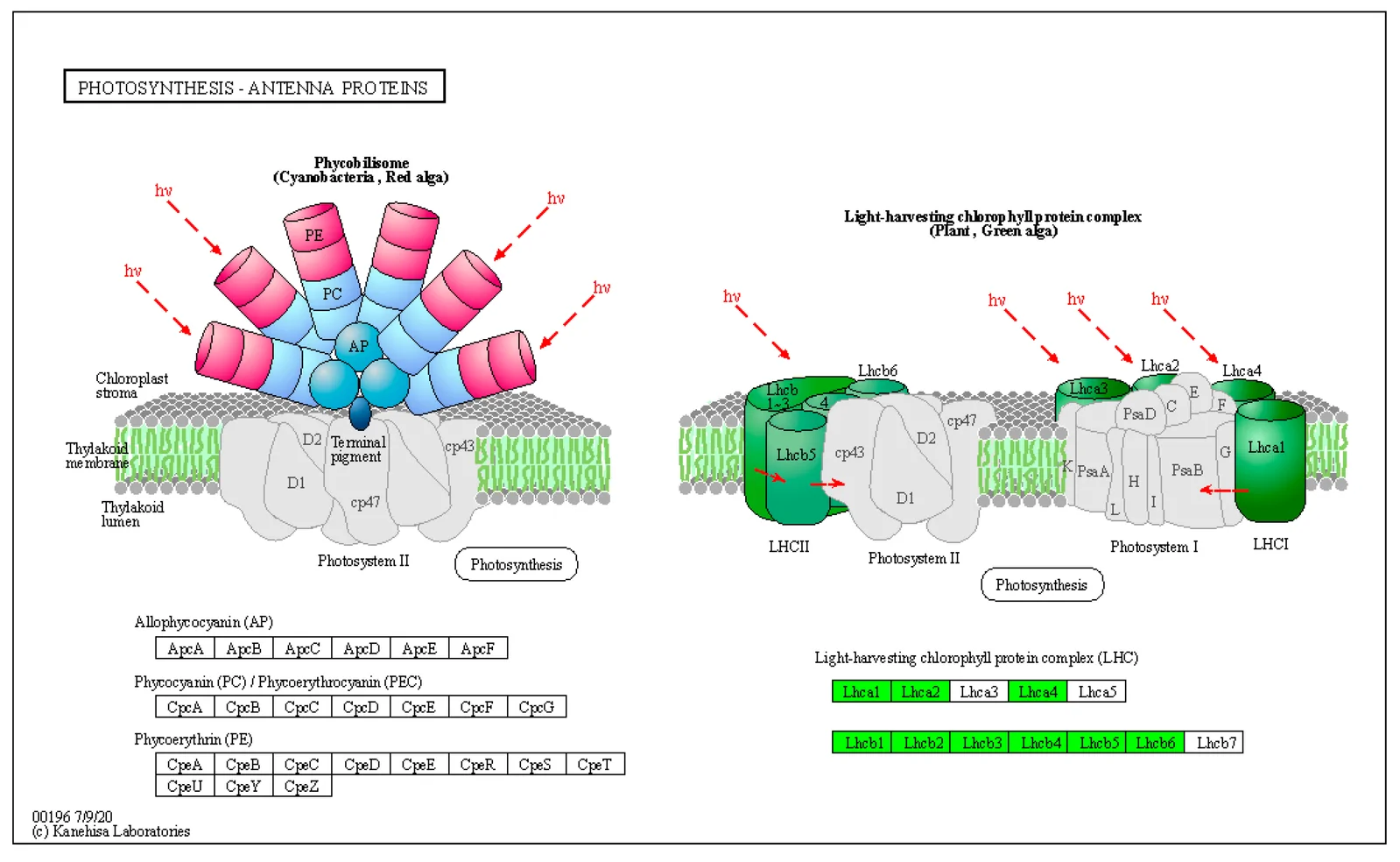
KEGG pathway map of photosynthesis–antenna proteins. Green-labeled components indicate genes with downregulated expression under hormone treatments. The pathway covers core components of light-harvesting chlorophyll protein complexes (LHCs) in photosystem I and photosystem II, as well as phycobilisome-related subunits.

Two computationally predicted hub candidates (*LOC104597786*, *LOC104598101*) were retrieved from this STRING-derived network. These two genes encode light-harvesting chlorophyll a/b-binding proteins (LHCs), whose canonical function is light-energy capture and excitation-energy transfer to PSI and PSII to maintain photosynthetic performance. It should be stressed that these genes represent only network-predicted candidate nodes; their biological functions and regulatory roles in hormone-mediated stress responses in lotus still require direct experimental validation. KEGG enrichment analysis revealed that both hub genes were enriched in the photosynthesis–antenna proteins pathway (Figure 7) and exhibited a consistent downregulated expression trend (*Lhca4*) under all three hormone treatments. These results indicate that distinct hormone signals may jointly suppress the expression of genes related to photosynthetic antenna proteins, forming a conserved molecular response mechanism.

Previous studies have demonstrated that LHC family genes are not only involved in light harvesting but also play vital roles in plant stress responses and the regulation of hormone signaling [38]. Upon external stimuli or hormone induction, downregulation of antenna protein-related genes may reduce light absorption efficiency, potentially alleviating photoinhibition and reactive oxygen species (ROS) accumulation triggered by excess light energy, thereby contributing to cellular redox homeostasis, and improves plant environmental adaptability. In the present study, the two hub genes displayed coordinated downregulation under the three hormone treatments. This pattern implies that diverse hormone-triggered transcriptional programs may converge on photosynthetic regulatory networks to adjust photosynthetic output and reallocate energy toward stress-adaptation responses. Still, transcript-level down-regulation does not guarantee corresponding changes in protein abundance or pathway activity. Therefore, the photosynthesis–antenna proteins pathway represents a promising candidate node for hormone-signal crosstalk, yet functional validation remains necessary.

**Figure 8:**
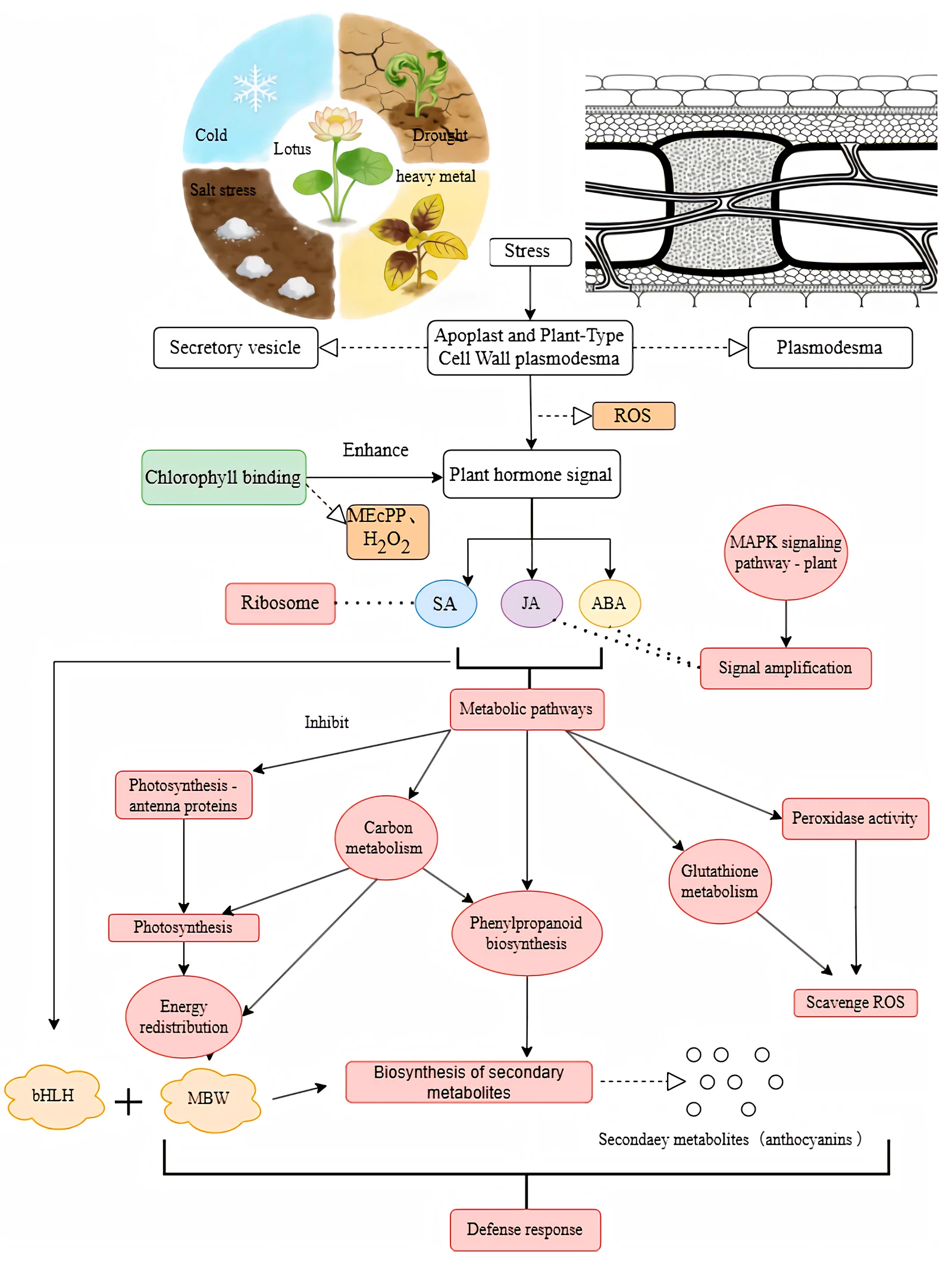
Proposed conceptual model of SA, JA and ABA responses in *Nelumbo nucifera* seedlings. The model was constructed based on transcriptome profiling results. Exogenous SA, JA and ABA signals may be perceived and transmitted through plant hormone signaling pathways, and may further influence downstream functional pathways including MAPK signaling, photosynthesis, secondary metabolite biosynthesis and ROS scavenging. These potentially interconnected pathways may contribute to stress response, energy redistribution and defense response in lotus seedlings [7,43,44,45].

Combined with previously reported findings, this study proposes a hypothetical conceptual model of plant PGRs in *Nelumbo nucifera* (Figure 8), which is built entirely on transcriptomic profiles. This study hypothesizes that when *Nelumbo nucifera* perceives external stress, the cell membrane generates ROS, which may facilitate signal transmission via the apoplast and symplast pathways, as well as through intracellular secretory vesicles. Meanwhile, chlorophyll binding may be associated with the regulation of MEcPP and H_2_O_2_, which may further contribute toward triggering of the plant hormone signaling network [39,40]. At the metabolic level, the three hormones converge on multiple shared physiological modules, including secondary-metabolite biosynthesis, ROS homeostasis, and photosynthetic reprogramming for energy reallocation. Previous evidence indicates that the bHLH transcription factor family participates in stress adaptation and can form the MYB-bHLH-WD40 (MBW) complex to modulate flavonoid and anthocyanin biosynthesis [41,42]. Although this model synthesizes our transcriptomic observations together with published knowledge, it remains a purely hypothetical framework. None of the proposed signaling cascades have been functionally validated in lotus, and further experimental work is required to verify these predicted regulatory relationships.

Despite these findings, several limitations of this study should be acknowledged. First, this research is mainly based on transcriptome data analysis, with insufficient experimental validation. Second, although nine topological analysis methods were adopted to improve the accuracy of hub gene screening, the results may still be affected by experimental conditions, sample sources, and species-specific biological characteristics. Third, only a single concentration and a single 24 h sampling time point were used for each exogenous hormone treatment; we did not set combined hormone treatment groups. In addition, direct quantification of endogenous hormones and related metabolites was not performed, and subsequent stress challenge experiments were lacking. Finally, the biological functions and regulatory mechanisms of the hub genes identified in the three hormone signaling pathways need to be further verified through in vitro and in vivo experiments. For future research, hormone induction experiments with different concentrations and treatment durations can be designed, combined with multi-omics approaches, to further elucidate the spatiotemporal characteristics of hormonal regulation in Nelumbo nucifera. Simultaneously, techniques such as gene cloning, overexpression, and knockout can be employed to validate the functions of the core co-responsive genes identified in this study and to clarify their mechanisms of action in the stress resistance process of Nelumbo nucifera. Overall, this study performs hypothesis generation and exploratory bioinformatics analysis based on transcriptome datasets, which can provide a preliminary theoretical basis for subsequent in-depth mechanistic research.

## 4. Materials and Methods

### 4.1. Plant Materials

The present study utilized the *Nelumbo nucifera* cultivar ‘Taikong’ as the experimental material. Healthy and plump seeds of *Nelumbo nucifera* were selected, and following mechanical scarification of the seed coat, they were cultivated hydroponically in distilled water within a greenhouse maintained at a constant temperature of 20 °C. Once the seeds germinated and the floating leaves had fully expanded, exogenous hormone treatments were applied. Seedlings exhibiting uniform growth were randomly assigned to four groups: a blank control group (CK), a salicylic acid treatment group (SA), a jasmonic acid treatment group (JA), and an abscisic acid treatment group (ABA). Each group comprised three biological replicates, with each replicate consisting of ten uniform seedlings. For each biological replicate, apical buds and young leaves from the ten seedlings were equally mixed before RNA extraction. The treatment groups were subjected to whole-plant immersion in solutions containing 500 μmol/L SA, 50 μmol/L JA, or 50 μmol/L ABA, respectively, while the control group was treated with an equivalent volume of distilled water for 24 h. The apical 5 cm shoot buds and young leaves of seedlings in each group were sampled for subsequent total RNA extraction [17].

### 4.2. Total RNA Extraction and Transcriptome Sequencing

Total RNA was isolated from samples of *Nelumbo nucifera* in each group using the TRIzol Total RNA Extraction Kit, and cDNA libraries were subsequently constructed. The libraries were then sequenced on the Illumina HiSeq sequencing platform. Transcriptome sequencing was performed by Wuhan Qingke Biotechnology Co., Ltd. (Wuhan, China) [46].

### 4.3. RNA-Seq Data Quality Control, Genome Alignment, Expression Quantification, and Differential Expression Analysis

Raw image data generated by high-throughput sequencing on the Illumina HiSeq platform were subjected to base calling using CASAVA software (version 1.8.2) to generate raw sequencing reads (Raw Data). The sequencing output was subsequently converted into FASTQ format [47]. Quality control and filtering of the raw reads were performed using fastp v0.20.0. The resulting high-quality filtered reads were then aligned to the reference genome of *Nelumbo nucifera* using HISAT2 v2.2.1 (https://www.ncbi.nlm.nih.gov/datasets/genome/GCF_000365185.1/, accessed on 31 August 2026) [48]. The genome files and corresponding reference annotation files can be obtained from (https://www.ncbi.nlm.nih.gov/datasets/genome/GCF_000365185.1/, accessed on 31 August 2026). SAMtools v1.10 was used to process the resulting alignment files. Gene expression levels were quantified using StringTie v2.0.4 [49], and expression values were normalized using both FPKM (Fragments Per Kilobase of transcript per Million mapped reads) and TPM (Transcripts Per Million) methods [50].

Gene-level raw read counts generated from the expression quantification step were used as input for DESeq2 v1.26.0 for differential expression analysis. Multiple testing correction was performed using the Benjamini–Hochberg procedure. Genes with an absolute fold change ≥ 2 and an adjusted *p*-value (*p*-adjust) < 0.05 were designated as significantly differentially expressed, including both upregulated and downregulated transcripts. Subsequently, Gene Ontology (GO) enrichment analysis and Kyoto Encyclopedia of Genes and Genomes (KEGG) pathway enrichment analysis were performed on the upregulated and downregulated DEGs to identify the primary biological functions and pathways affected by the transcripts [51,52]. Subsequently, gene annotations were compared and integrated using the bioinformatics cloud platform (https://www.tsingke.com.cn/, accessed on 31 August 2026).

### 4.4. Functional Enrichment Analysis of DEGs

Gene Ontology (GO) enrichment analysis was performed using the GOseq R package based on the Wallenius non-central hypergeometric distribution. KEGG pathway enrichment analysis was performed using KOBAS. All screened DEGs were mapped to the GO database (http://www.geneontology.org/, accessed on 31 August 2026) and the KEGG database (https://www.kegg.jp/, accessed on 31 August 2026) for enrichment analysis. The number of DEGs associated with each term was calculated, and statistical tests were subsequently applied to identify terms that were significantly enriched among the DEGs compared with the whole-genome background. A threshold of adjusted *p*-value (*p*-adjust) ≤ 0.05 was applied to define significantly enriched terms. The significantly enriched results were visualized using TBtools-II and the Bioinformatics Online Platform (https://www.bioinformatics.com.cn/, accessed on 31 August 2026) [53].

### 4.5. Construction of Protein–Protein Interaction (PPI) Network and Identification of Hub Genes

To explore the potential interaction relationships among genes involved in different hormone response pathways, the protein sequences of the 607 shared DEGs were subjected to BLASTX-based sequence similarity searches against the genome of a related species with available protein–protein interaction information in the STRING database (https://cn.string-db.org/, accessed on 31 August 2026) [54]. The minimum interaction confidence score was set to 0.7, and interaction data were exported after removing isolated nodes. Subsequently, the resulting PPI network was imported into Cytoscape v3.10.4 for visualization and network analysis [55]. Hub genes were identified using the cytoHubba plugin in Cytoscape v3.10.4. Nine topological algorithms, including MCC, MNC, Degree, EPC, Closeness, Betweenness, BottleNeck, Stress, and Radiality, were applied. The top 10 genes ranked by each algorithm were selected, and genes consistently identified across the nine algorithms were defined as hub genes.

### 4.6. Validation by Quantitative Real-Time PCR

To verify the reliability of transcriptome data, 14 genes were randomly selected from DEGs (Supplementary Table S3), and quantitative real-time PCR (qRT-PCR) was used to detect their expression profiles under each treatment. The primer sequences are listed as follows (Supplementary Table S5).

Total RNA used for qRT-PCR was reverse-transcribed into cDNA of lotus samples following the manufacturer’s instructions of the reverse transcription kit (Vazyme, Nanjing, China). A volume of 2 µL cDNA was used as the template to establish a 15 µL qRT-PCR reaction system, containing 7.5 µL SupRealQ Purple Universal SYBR qPCR Master Mix, 0.3 µL of 10 µM forward primer, 0.3 µL of 10 µM reverse primer and 4.9 µL ddH_2_O. Three technical replicates and three biological replicates were set for each treatment [56]. LOC104593066 was used as the internal reference gene for normalization of target gene expression in the qRT-PCR analysis. The relative expression levels of the target genes were calculated using the 2^−ΔΔCt^ method.

### 4.7. Statistical Analysis

Statistical analyses were conducted using GraphPad Prism 9.0. Figures were visualized and generated with Prism (v9.0), TBtools-II, R software (version 4.5.1), and the Bioinformatics Online Platform (https://www.bioinformatics.com.cn/, accessed on 31 August 2026). Statistical significance was conventionally defined as *p*-value < 0.05. In this study, statistical analysis was performed using *p*-values adjusted for multiple testing via the Benjamini–Hochberg (BH) procedure, designated as *p*-adjust.

## 5. Conclusions

In this study, seedlings of the *Nelumbo nucifera* cultivar ‘Taikong’ were used as experimental material. Through transcriptome sequencing, we systematically elucidated that the transcriptional regulation of *Nelumbo nucifera* seedlings by SA, JA, and ABA exhibits significant functional specificity, with each hormone forming a regulatory pattern adapted to its core stress-resistance function. The regulation of gene expression in *Nelumbo nucifera* seedlings by SA was predominantly characterized by global repression, with regulatory targets deeply covering photosynthetic system structure and ribosomal translation pathways. The core strategy involved reallocating energy toward defense pathways by suppressing basal growth and metabolic processes such as photosynthesis, which represents the central regulatory strategy by which SA mediates stress responses in *Nelumbo nucifera*. The regulatory patterns of JA and ABA were more similar; DEGs responding to both hormones were enriched in stress-signal-related gene sets, pointing to potential involvement of plant hormone signal transduction and MAPK signaling modules. Among them, JA-responsive genes were mainly associated with defense responses, whereas ABA-responsive genes were enriched in processes related to photosynthetic metabolism and stress signal transduction, consistent with its established role in plant stress responses.

This study identified a total of 607 core genes showing shared transcriptional responses to SA, JA, and ABA treatments. These genes were predominantly enriched in cold response, defense response, metabolism, and secondary metabolite biosynthesis pathways, highlighting the involvement of photosynthesis-, metabolism-, and defense-related processes in the responses of Nelumbo nucifera seedlings to hormone treatments and stress. Furthermore, transcriptomic data suggest the apoplast as a potential compartment for multi-hormone stress-signal convergence in *Nelumbo nucifera*. Moreover, the photosynthesis–antenna proteins pathway emerges as a promising candidate node for potential hormone-signal crosstalk. Two LHC-family genes stand out as computationally predicted hub candidates from PPI-network analysis, and their in vivo functions remain to be experimentally validated. These findings provide novel theoretical insights into the molecular mechanisms by which PGRs regulate photosynthesis and environmental adaptation, uncover the broad-spectrum molecular basis of stress tolerance in *Nelumbo nucifera* under complex environmental conditions, and offer core candidate targets for screening broad-spectrum stress-resistant genes in *Nelumbo nucifera*.

## Data Availability

The transcriptome data are deposited in NCBI under the accession number PRJNA1503730.

## Supplementary Materials

The following supporting information can be downloaded at https://www.mdpi.com/article/10.3390/ijms27177957/s1.
